# Frailty is Associated with an Increased Risk of Major Adverse Outcomes in Elderly Patients Following Surgical Treatment of Hip Fracture

**DOI:** 10.1038/s41598-019-55459-2

**Published:** 2019-12-13

**Authors:** Chiu-Liang Chen, Chun-Min Chen, Chun-Yi Wang, Po-Wei Ko, Chung-Hwan Chen, Chen-Pu Hsieh, Herng-Chia Chiu

**Affiliations:** 1 Department of Orthopedic Surgery, Changhua Christian Hospital, Changhua, Taiwan; 2 Research Education and Epidemiology Center, Changhua Christian Hospital, Changhua, Taiwan; 3 Department of Orthopedic Surgery, Yuanlin Christian Hospital, Changhua, Taiwan; 4 Orthopedic Research Centre, Kaohsiung Medical University, Kaohsiung, Taiwan; 5 Department of Orthopedics, Kaohsiung Municipal Ta-Tung Hospital, Kaohsiung Medical University, Kaohsiung, Taiwan; 6 Institute of Hospital Management, Tsinghua University, Beijing, China; 7 Department of Healthcare Administration and Medical Informatics, Kaohsiung Medical University, Kaohsiung, Taiwan; 8 Department of Health Policy and Management, Bloomberg School of Public Health, John Hopkins University, Baltimore, Maryland United States of America

**Keywords:** Preventive medicine, Risk factors

## Abstract

We intended to explore the effect of level of frailty on, and relationship with, 1-, 3-, and 6-month postoperative emergency department visits, readmissions, and mortality. This is a prospective multicenter observational cohort study design. Patients aged 50 years or older treated for hip fracture (*n* = 245) were taken from Orthopedic wards in one medical center (*n* = 131) and one district hospital (*n* = 114) in Changhua County, Taiwan. Frailty was defined as measured by the validated Clinical Frailty Scale and categorized as robust, pre-frail, and frail. We used Kaplan-Meier analysis to estimate survival rates and Cox regression to estimate the risk of frailty associated with adverse outcomes. To examine the longitudinal associations between frailty and adverse outcome, the cross-lagged models were explored. Of the 245 patients, 55 (22.4%) were classified as frail, 113 (46.1%) as pre-frail, and 77 (31.4%) as robust. More cumulative events occurred for frail than for robust patients for each adverse outcome. Frailty has long-term effect on each adverse outcome after discharge, rather than the effect simultaneously. Targeting pre-frailty and frailty is essential for prevent adverse outcomes and improving the overall health of older adults after discharge for hip fracture.

## Introduction

Hip fracture is a major public health issue. Cheung *et al*.^1^ predicted that the total number of hip fractures in Asian countries will increase from 1.12 million in 2018 to 2.56 million in 2050, and direct medical costs will increase from $9.5 billion in 2018 to $15 billion in 2050. In Taiwan, with improved medical quality, the mortality rate after hip fracture has been declining, but the annual incidence of hip fracture has continued to increase, from 405/100,000 to 463/100,000^2,3^. Hip fractures are associated with morbidity and loss of independence, and adverse clinical outcomes after hospital discharge are common and costly occurrences^4–6^. Most studies have attempted to identity risk factors for adverse events^7^ and to explore outcomes such as mortality, length of inpatient stays, and mobility^4,8^ after hip fracture surgery. However, various adverse consequences for hip fracture patients at different time points after surgery remain unknown^9^. Identification of these interactions is important for future initiatives to reduce adverse outcomes by targeting resources toward the higher risks at the proper time.

Frailty has been shown to predict adverse outcomes in older surgical patients^8–13^. Compared with low frailty levels, higher degrees of frailty lead to greater risk during the postoperative period, with more postoperative complications^14^, longer lengths of stay^8^, early readmission^10,11^, and greater morbidity and mortality^15^. However, most studies have focused exclusively on patients with severe frailty^8,13^, and outcomes for patients at early frailty stages (pre-frailty) remain unclear. This study examined frailty and pre-frailty statuses in predicting hip fracture outcomes to understand the magnitude of adverse events associated with different stages of frailty.

Among all health care challenges, the impact of frailty on health and health care outcomes is of great importance, while the nature and temporality of the relationship between frailty and adverse outcome (e.g. mortality, first readmission, emergency department visit) remain elusive, and no study has examined their temporal relationships after hip fracture. We therefore developed a model that situates adverse outcomes after hip fracture at the intersection of an individual’s frailty state and risk factors. We hypothesized that: 1) frailty and adverse outcome would be strongly associated throughout the 6 months post-hip fractur surgery; and 2) the previous frailty state would be the stronger contributor to sequent frailty and adverse outcomes. To test the hypothesis, the study aimed to evaluate the magnitude of adverse outcomes at different frailty levels at 1-, 3- and 6-months post-hip fracture using a survival analysis; to examine long-term relationships between frailty and each adverse outcome at 1-, 3- and 6-months post-hip fracture using a cross-lagged panel analysis structural equation model (SEM), which examines the structural relationships of repeatedly measured constructs.

## Methods

### Data collection and follow-up

This was an observational cohort study with observation periods from 2017 to 2018 at the department of orthopedics at one medical center and one regional hospital in central Taiwan. This study has adopted a convenience sampling approach (non-probability sampling). We chose the studying hospitals because they are in the same medical service area and are in the umbrella of our health system which enable us to collect the clinical information and complete the follow-up surveys. Only hip fracture patients (ICD-10-CM S72.0* S79.0*) who underwent surgical treatment for hip fracture between January 2017 and January 2018 were enrolled (*n* = 277). We excluded patients aged younger than 50 years, with poor cognitive function, whose injury was not caused by low-energy trauma, who had primary or secondary tumor-related fractures, who were wheelchair restrained or permanently bedridden before fracture, who died preoperatively, whose information was incorrect or incomplete in the electronic medical record (EMR), or who were lost to follow-up.

The study group consisted of 166 women and 79 men with a mean age of 78 (range, 53–97) years at the time of surgery. In total, the preoperative diagnosis was femoral neck fracture (non-displaced) for 14 (5.7%), femoral neck fracture (displaced) for 100 (40.8%), intertrochanteric fracture for 103 (42.0%), and subtrochanteric fracture for 28 (11.4%) patients. Osteosynthesis was performed for 140 (57.1%) fractures using percutaneous cannulated screws (14 fractures), sliding hip screws (14 fractures), or an intra-medullary nail (proximal femoral nail, 112 fractures). Bipolar hemiarthroplasty was performed for 105 fractures (42.9%). Non-clinical information was collected from the patient (when cognitive performance was intact) or from a caregiver as proxy.

Patient clinical information was obtained from the hospital’s EMR system by experienced charters. This study protocol was approved by Human Research Protection Program of Changhua Christian Hospital and adhered to the principles of the Declaration of Helsinki. Informed consent was obtained from all participants. The study was approved by the Changhua Christian Hospital institutional review board (IRB No:160703).

### Outcome variables

One-, 3-, and 6-month outcomes, including mortality, emergency department visits, and readmissions, were the main outcome variables. All-cause mortality was defined as the primary outcome. Readmission and emergency department visits were defined as patients admitted to participating hospitals or emergency departments (ED) due to postoperative complications (such as a wound infection, delirium, pneumonia, heart failure and pressure ulcers)^16–18^, unplanned readmission within 6 months after discharge. All outcomes data during the 180-day follow-up period were confirmed by the EMR system.

### Assessment of frailty

We assessed frailty using the Chinese-Canadian Study of Health and Aging Clinical Frailty Scale (CSHA-CFS), validated in previous studies^19,20^. The index is simple and easy to use, predicting health and aging through rapid screening for potentially frail older adults. The frailty scale scores ranged from 1 (very fit) to 7 (severely frail). Each level is defined as follows: Level 1: very fit and robustly active; Level 2: well without significant disease complications; Level 3: well with controlled disease; Level 4: no obvious dependency, but vulnerable; Level 5: mildly frail, with limitations in instrumental activities of daily living (IADLs); Level 6: moderately frail and requiring assistance for ADLs and IADLs; Level 7; severely frail and completely dependent on others for ADLs. Baseline frailty was surveyed by face-to-face interview, and patients were assessed for preoperative frailty before discharge. Based on the CHS phenotypic definition of frailty^21^, frailty status was categorized as robust (Levels 1–3), pre-frail (Levels 4–5), and frail (Levels 6–7).

### Other covariates

Age, sex, marital status, cognitive function, body mass index (BMI)^22^, comorbid conditions, Charlson Comorbidity Index (CCI)^23^, fracture type, implant type applied during surgery, preoperative time to surgery, and total length of postoperative hospital days were covariates at the analyses.

### Statistical analysis

Descriptive analyses (mean, SD, and percentage) were conducted for demographic and clinical data to characterize the sample. A correlation matrix was run to assess relationships between all indicator variables (frailty and adverse outcomes at 1-, 3- and 6-months post-hip fracture surgery) prior to model creation. Relation status between categorical variables was assessed using the chi-square test. The two-sample t-test was implemented to compare independent variables. The survival analysis was adopted to estimate mortality, readmissions, and emergency department (ED) visits admission rates after surgery for hip fracture. Overall survival rates were estimated using Kaplan-Meier (K-M) analysis. The log-rank test and multivariate Cox proportional hazards model were applied to estimate the effects of risk factors on survival time.

To examine the longitudinal associations between frailty and adverse outcomes, the cross-lagged models were explored based on structural equation modeling (SEM)^24^. SEM allows for multiple relationships to be analyzed simultaneously, allowing the user to build more complex statistical models rather than running several linear regressions. The cross-lagged design comprises two or more variables at two or more time points. It yields three types of effects: synchronous associations (correlations between different variables measured at the same time), stability effects (correlations between the same variable measured at different times), and cross-lagged effects. The relative strengths of longitudinal relationships can be determined through comparison of standardized betas.

To deal with missing cases, we used imputation process of the maximum likelihood estimation in the variables of interest. Thus, we carried out the statistical analyses with 245 patients at 3 time points of the study. The maximum likelihood estimation is probably the most pragmatic missing data estimation approach for SEM. It has shown evidences of unbiased parameter estimates and standard errors for data missing at random and data missing completely at random^25^. The model fit was evaluated using several indices: the comparative fit index (CFI), the root means square error of approximation (RMSEA), the Tucker–Lewis Index (TLI), and the Normed Fit Index (NFI). Values of CFI ≧ 0.90, TLI ≧ 0.90 RMSEA < 0.08 are assumed to be indicative of a good-fitting model, as recommended by Hu and Bentler^26^ and McDonald^27^. IBM SPSS Statistics for Windows and AMOS, version 22.0 (IBM Corporation., Armonk, NY, USA) was used to analyze the results. The level of significance was set at *P* < 0.05.

## Results

Table 1 summarizes the clinical characteristics of patients by frailty status. Frailty level does not significantly differ between the two research hospitals. Prevalence of pre-frailty and frailty was much higher for women than for men. Overall, frail patients were significantly older, with lower BMI and worse cognitive functioning than pre-frail or robust patients. Marital status, fracture type, and implant type did not differ significantly by frailty group. Comparisons of cognitive functioning in the past year showed an increasing trend with frailty levels. Frail elderly patients had the highest CCI scores, followed by robust and pre-frail subjects. Comparisons of time to surgery and postoperative hospital stay showed no statistical differences between patients by frailty status.

**Table 1:** Characteristics of the study group by frailty status.

		Robust		Pre-frail		Frail		P
		n	%	n	%	n	%	
Hospital	Medical center	40	52%	62	55%	29	53%	0.917
	Regional hospital	37	48%	51	45%	26	47%	
Age, years	≤75	52	68%	26	23%	6	11%	<0.001
	76–85	22	29%	52	46%	27	49%	
	≥86	3	4%	35	31%	22	40%	
Sex	Male	36	47%	27	24%	16	29%	0.004
	Female	41	53%	86	76%	39	71%	
Marital status	Alone	21	27%	48	42%	25	45%	0.050
	With spouse	56	73%	65	58%	30	55%	
Cognitive function	Normal	74	96%	82	73%	29	53%	<0.001
	Mild impairment	2	3%	15	13%	10	18%	
	Moderate to severe	1	1%	16	15%	16	29%	
BMI	Under/normal weight	33	43%	62	55%	33	60%	0.072
	Overweight	17	22%	19	17%	14	25%	
	Obese	27	35%	32	28%	8	15%	
CCI	None	28	36%	45	40%	15	27%	0.354
	1–2	31	40%	44	39%	21	38%	
	≥3	18	23%	24	21%	19	35%	
Comorbidities	none	45	58%	76	67%	36	65%	0.448
	≥1	32	42%	37	33%	19	35%	
Fracture type	Femoral neck fracture (undisplaced)	7	9%	4	4%	3	5%	0.613
	Femoral neck fracture (displaced)	34	44%	44	39%	22	40%	
	Intertrochanteric	27	35%	53	47%	23	42%	
	Subtrochanteric	9	12%	12	11%	7	13%	
Implant type	Cannulated screws	7	9%	4	4%	3	5%	0.367
	Sliding hip screw	5	6%	6	5%	3	5%	
	Intra-medullary nail (Gamma nail)	27	35%	58	51%	27	49%	
	Bipolar hemiarthroplasty	38	49%	45	40%	22	40%	
Mean time to surgery(days), (Mean ± SD)		8.1 ± 22.4		4.0 ± 10.1		3.8 ± 8.3		0.125
Postoperative hospital stay(days), (Mean ± SD)		7.2 ± 3.8		7.1 ± 2.5		7.0 ± 3.5		0.885

BMI, body mass index; CCI, Charlson Comorbidity Index.

**Figure 1:**
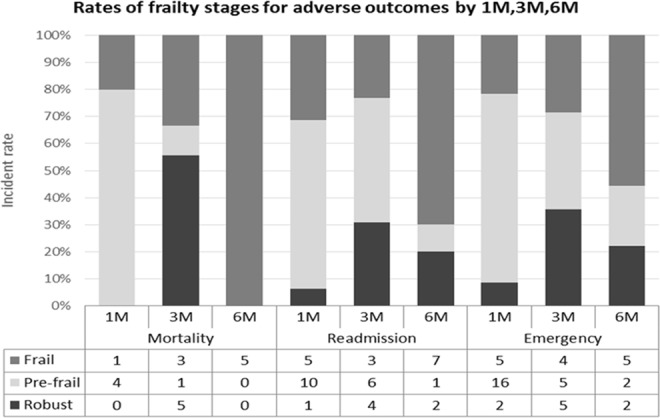
Rates of mortality, readmission, and emergency department visits for robust, pre-frail and frail patients at 1, 3, and 6 months. Adverse outcomes tended to vary by frailty level.

The three time points were 1, 3 and 6 months all-cause mortality, all-cause hospital readmission, and all-cause ED visits after surgery. Figure 1 outlines the proportions of patients classified as robust, pre-frail, and frail according to each outcome at 1, 3, and 6 months. All-cause mortality and readmission rates in frail patients increased over time, with a peak period of six months after surgery. Conversely, pre-frail patients returned to hospital for emergency and readmission rates are higher within one month after surgery. Surprisingly, the deterioration of the robust group occurred intensively within three months after surgery.

**Figure 2:**
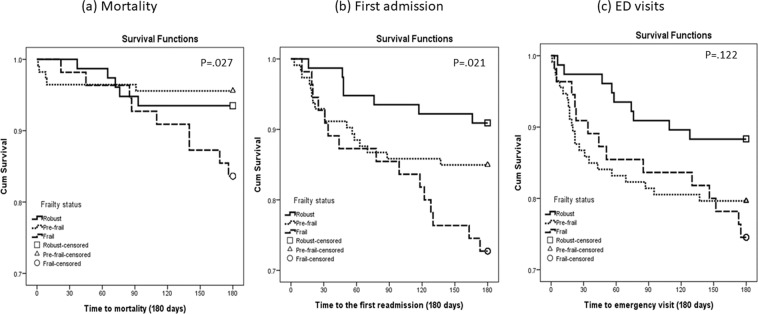
Cumulative survival curves for (**a**) mortality, (**b**) readmission, and (**c**) emergency department visits by frailty level. Kaplan-Meier curves for cumulative 6-month all-cause survival by frail, pre-frail, and robust status. Log-rank test shows statistically significant difference between the three groups for mortality and readmission.

The cumulative survival rates of the frailty groups were statistically significant different from the other groups according to the K-M curve (*P* < 0.05 by log-rank test), especially for higher mortality and readmission (Fig. 2). Six months after discharge, the survival curves for frail, pre-frail, and robust patients differed; cumulative survival rates for frail and pre-frail patients were significantly lower than rates for robust patients. The magnitude of difference was greater for mortality and readmission. Overall, cumulative survival curves for frail and pre-frail patients for mortality, readmission, and ED visits showed decreasing trajectories over time.

Table 2 shows the risk of adverse outcomes after hip fracture associated with frailty and pre-frailty at different time points. After adjustment, compared with the robust group, the frail patients had a higher risk of 6-month mortality and readmission, and the pre-frail patients had a higher risk of 3-month readmission and 1-month ED visits (Table 2). For mortality, hazard ratios (HRs) for frail patients ranged from 4.9 (in Model 1), 4.8 (in Model 2) to 4.6 (in Model 3) times higher than robust patients, 6-month after discharge. Although the mortality rate in the robust group was higher at the first 3-month after surgery than in the either frail or pre-frail group, there was no significant difference between the two groups after adjustment by different models. For readmission, HRs for pre-frail patients were 3.16 (M1), 3.37 (Model 2) to 3.06 (M3) times higher than for robust group 3 months after discharge; HRs were 4.31(Model 1) to 4.38 (Model 2) times higher for frail patients 6 months after discharge. In addition, pre-frail patients were 8.48 (Model 1), 8.91 (Model 2) to 8.99 (Model 3) times more likely to have ED visits 1 month after discharge; HRs for pre-frail were 2.85 (Model 1) to 2.86 (Model 2) times higher 3 months after discharge. The results revealed that both frailty and pre-frailty were significant risk factors for adverse outcomes.

**Table 2:** Outcomes within 1, 3, and 6 months after discharge, by frailty status.

		Model 1			Model2			Model3		
		(Adjusted for age and sex)			(Adjusted for age, sex, cognition, BMI)			(Adjusted for age, sex, cognition, BMI, CCI, and comorbidities)		
		HR	95.0% CI	P value	HR	95.0% CI	P value	HR	95.0% CI	P value
**Mortality**										
1M	Pre-frail	NA	NA-NA	—	NA	NA**-**NA	—	NA	NA**-**NA	—
	Frail	NA	NA-NA	—	NA	NA**-**NA	—	NA	NA**-**NA	—
3M	Pre-frail	1.31	(0.29–5.98)	0.723	1.23	(0.27–5.64)	0.787	1.27	(0.27–5.97)	0.762
	Frail	2.96	(0.61–14.50)	0.180	2.56	(0.48–13.56)	0.270	1.67	(0.27–10.30)	0.578
6M	Pre-frail	1.17	(0.31–4.46)	0.814	1.21	(0.31–4.64)	0.780	1.21	(0.31–4.79)	0.787
	Frail	4.90	(1.40–17.10)	0.013	4.80	(1.29–17.82)	0.019	4.60	(1.05–20.14)	0.043
**Readmission**										
1M	Pre-frail	6.82	(0.77–60.58)	0.085	6.46	(0.70–59.88)	0.100	6.40	(0.67–60.78)	0.106
	Frail	7.20	(0.69–75.52)	0.100	6.25	(0.54–72.06)	0.142	3.85	(0.29–50.81)	0.305
3M	Pre-frail	3.16	(1.07–9.37)	0.038	3.37	(1.12–10.10)	0.030	3.06	(1.01–9.30)	0.049
	Frail	3.36	(0.98–11.54)	0.055	3.16	(0.87–11.42)	0.079	1.87	(0.50–7.01)	0.355
6M	Pre-frail	2.21	(0.85–5.74)	0.104	2.42	(0.91–6.41)	0.077	2.21	(0.83–5.92)	0.114
	Frail	4.31	(1.56–11.94)	0.005	4.38	(1.49–12.86)	0.007	2.96	(0.97–8.98)	0.056
**Emergency visit**										
1M	Pre-frail	8.48	(1.78–40.29)	0.007	8.91	(1.83–43.34)	0.007	8.99	(1.82–44.46)	0.007
	Frail	5.92	(1.00–34.85)	0.049	5.70	(0.91–35.51)	0.062	4.87	(0.77–30.97)	0.094
3M	Pre-frail	2.85	(1.10–7.40)	0.031	2.86	(1.09–7.52)	0.033	2.60	(0.98–6.93)	0.056
	Frail	2.39	(0.78–7.35)	0.129	2.05	(0.63–6.64)	0.231	1.44	(0.43–4.79)	0.551
6M	Pre-frail	2.36	(1.00–5.57)	0.050	2.31	(0.96–5.51)	0.060	2.10	(0.87–5.05)	0.098
	Frail	2.90	(1.10–7.64)	032	2.46	(0.89–6.79)	0.081	1.71	(0.61–4.82)	0.311

CI, confidence interval; HR, hazard ratio. NA, data not available.

Notably, age, marital status, education, cognitive status, BMI, CCI, fracture site and cause did not significantly differ between two research hospitals (data not shown). Differences in preoperative diagnosis and osteosynthesis performance were found, as a matter of fact, we included provider’s attribute and clinical practice as covariates for model testing; however, none of them was significantly associated any adverse outcome. Therefore, we removed these covariates from the final models.

**Figure 3:**
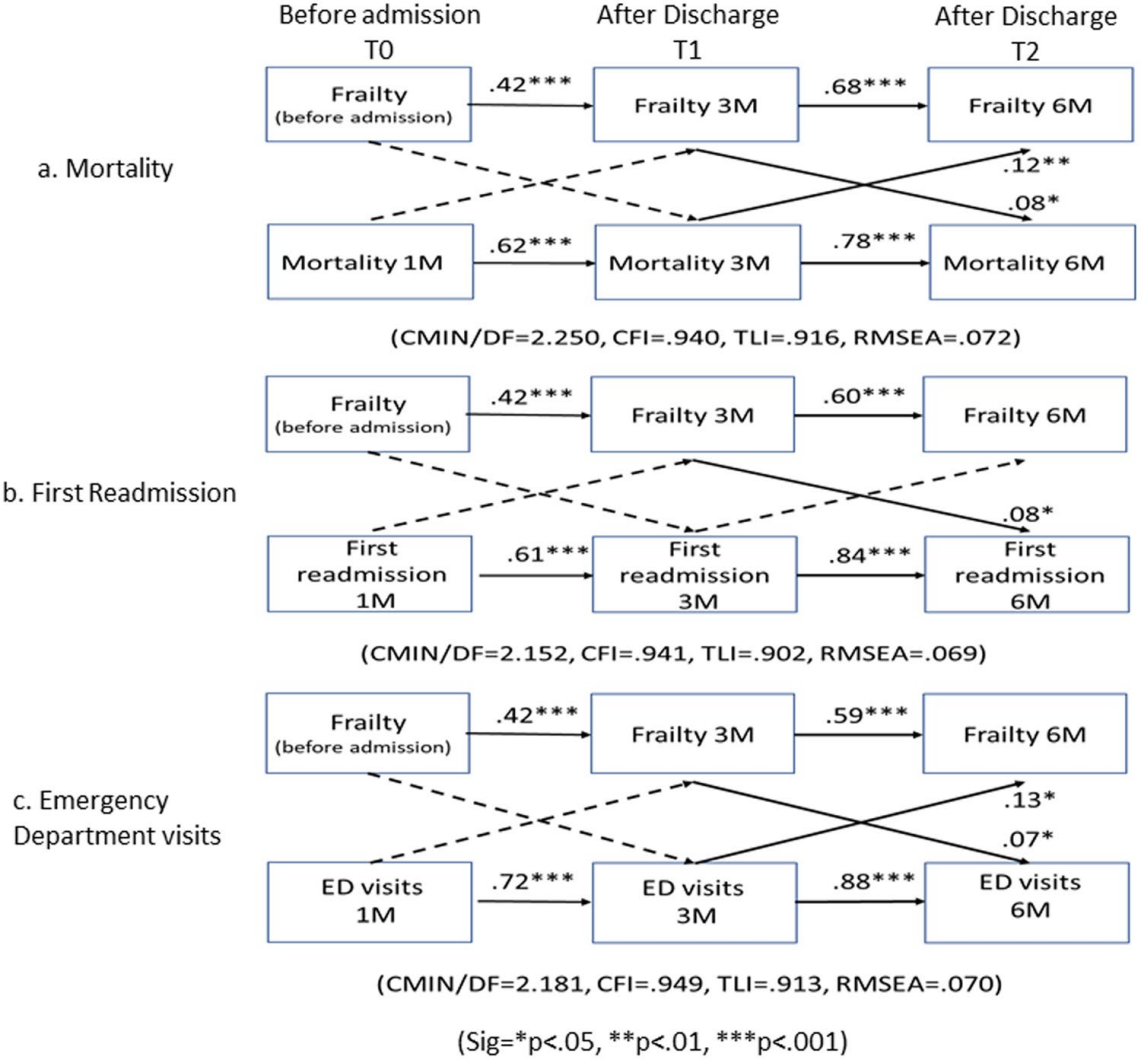
A pathway model of the effects of frailty on adverse outcome (mortality, first readmission, and ED visits) with time-constant predictors. Notes:1. Pathway models adjusted for age, sex, cognitive function, CCI, and BMI. 2. Single-headed arrows display hypothesized directional relationship. All of the paths were estimated. After controlling for covariates, some paths became nonsignificant and were presented as the dotted lines. The 17 solid-line pathways represent significant effects with standardized regression coefficients along the paths. 3. These models had satisfactory model fit in terms of values of CFI, TLI, and RMSEA.

The standardized regression coefficients from the reciprocal models are shown in Fig. 3, with adjustment for covariates. Figure 3(a–c) presents a final path diagram for frailty status and each adverse outcome. In Fig. 3a, the six solid-line pathways (four lag effects with horizontal arrows and two cross-lagged effects with diagonal arrows) represent significant longitudinal relationships between frailty and mortality. In Fig. 3b, the five solid-line pathways (four lag effects with horizontal arrows and one cross-lagged effect with diagonal arrow) represent significant longitudinal relationships between frailty and first readmission. In Fig. 3c, the six solid-line pathways (four lag effects with horizontal arrows and two cross-lagged effects with diagonal arrows) represent significant longitudinal relationships between frailty and ED visits.

Specifically, in longitudinal relationships, lag effects were found among all three models suggesting that frailty status at each time point predicts future status, this was also found in all health outcomes. Furthermore, the results support that the directions of these cross-lagged paths could be reciprocal. For example (Fig. 3c), patients with frailty at the time of 3 months (3 M) after discharge were more likely to experience in ED visits at 6 months (6 M) after discharge (0.07), on the other hand, patients with ED visits after 3 M discharge were more likely to experience deteriorations in frailty at 6 M after hospital discharge (0.13), and the relationships are indeed correlational in nature. The correlations between cross-sectional variables (e.g. Frailty 3 M ↔ mortality 3 M) were not found in the final path models, suggesting that frailty has long-term effect on adverse outcome after discharge, rather than the effect simultaneously. Collectively, these findings suggest a longitudinal relationship among frailty and mortality, first readmission and ED visits within 12 months post-discharge.

## Discussion

In the present study, we proposed a theoretical pathway in which frailty status is a well-known correlate of adverse outcome after hospital discharge, influenced mortality, first readmission, and ED visits for hip fracture older patients. Our findings indicate that older hip fracture patients’ health following hospital discharge is a complex process, frailty status and different adverse outcomes often temporally influencing each other. The main findings were: (1) the cumulative survival curves for frailty for each outcome showed decreasing trajectories; (2) frailty and each adverse outcome show lagged effects on subsequent measures in the same domain; (3) effects of previous frailty on subsequent adverse outcome and vice versa further suggest a reciprocal interrelationship; (4) frail patients were more likely than robust patients to die within 6 months after discharge; (5) pre-frail patients were more likely than robust patients to have early ED visits (1 month) and readmissions (3 months). The longitudinal relationships between variables (i.e. all solid lines) are substantiated in the models, and that detecting frailty status can help to prevent adverse outcome in the post-discharge phase.

In fact, awareness of frailty may cause adverse health outcome as nature reactions to population ageing. Frailty has been reported to be associated with short-term mortality^28^, our study indicates that older patients with frailty tend to have higher risk of 6-month mortality than those with pre-frail, which accounted for 47% of total deaths. In addition, frailty defined by CSHA-CFS was associated with a more than fourfold increase in 6-month mortality, which was comparable with a study of frailty defined by the Reported Edmonton Frail Scale (REFS) in older hospitalized patients^29^, suggest that CSHA-CFS is suitable for screening frailty and predicting mortality in acute care. A screening test for frailty, using a measure of frailty that is routinely available, has the potential to allow appropriate intervention and tailored treatment and finally can help prevent adverse outcomes.

Furthermore, frailty was associated with an increased risk of hospital readmission^30^. Our study found that the most common cause of readmission was respiratory disease among elderly trauma patients^14,31^, indicating that frail patients were more likely to develop pulmonary complications than non-frail patients. Also, the higher percentage of pneumonia (23%) observed in this group might be related to increased incidence of readmission events. As deteriorated frailty status may be an early sign rather than a risk factor for adverse outcome, the temporal relation between frailty and first readmission in older patients needs to be further tested.

Few studies have addressed ED visits following hip fracture surgery, although it is recognized as a quality matter. Emergency department (ED) visits for urinary tract infections (UTI) are common among the hip fracture patients^32–34^. Higher rates of pre-frailty were observed in clinical patients^35^, which was also confirmed by our study. The findings may actually reflect the association between the pre-frailty and the incident of the ED visits, as postoperative UTI (or fever) and the use of ER strongly related to each other. Therefore, for pre-frail patients, close observation is needed during postoperative hospitalization, as is a plan for follow-up after discharge.

Nevertheless, the presence of adverse heath outcome after discharge may negatively impact older patients’ subsequent frailty status. The inappropriate readmissions and unnecessarily long periods in hospital can be harmful and may mean that the elderly have deteriorated to a point where they can no longer return home. A study found that mortality increased by 43% after 10 days of admission due to an overcrowded ED^36^. Treating prior condition might improve another condition, early detecting frailty might improve health outcome so targeting this bidirectional link with an intervention along with necessary therapy might not only more effectively treat frailty symptoms but also improve overall function in older adults following an acute care for medical and surgical services. The weak effect of frailty on adverse outcome might be due to the small number of event cases. Since SEM is a large sample approach^37^, the small number of events (adverse outcomes) probably may lower the power of model fit.

There is currently no consensus regarding the best predictive markers for postoperative mortality and readmission after surgery in older hip patients. Although both the Charlson comorbidity index (CCI)^38,39^ and the frailty^29,40^ are known to be risk factors for postoperative outcomes, it is unclear which factor has a stronger predictive power for different outcomes. Our study found that increased CCI was associated with higher readmission and emergency rates after 1-, 3-, 6-months hip fracture surgery (data not shown). However, frailty is associated with higher mortality rates after 6 months of hip fracture surgery. Although the frailty was not designed to predict perioperative mortality in surgical cohorts, it may correlate with a greater risk than CCI for perioperative death in the elderly and need to pay more attention.

A major strength of our study is that it examined the course of mortality, first readmission, ED visits and their interrelationships with frailty status, adjusted for important covariates, in older patients with hip fracture from acute hospitalization to 6 months afterward. Although this was a prospective cohort study with objective assessment of adverse outcomes by observers of patient frailty status, it has some limitations. First, the frailty assessment was based on the patient’s or caregiver’s subjective impression of premorbid functioning. Information obtained from caregivers might not be an accurate assessment of individual patient responses. Second, our findings are representative of data from two institutions in the same county and may not generalize to other geographical areas. Third, although the potential associated factors were examined, the effects of non-observable factors (e.g. caregiver issue) cannot tested, which might improve the prediction of analytical modeling. Fourth, our categorization and identification of frailty status were based on CSHA-CFS, but not diagnostic interviews; this limitation was assumed to be is minimized by the widespread use of the frailty screening instrument to reliably measure frailty status^19^. Fifth, different clinical practice by hospitals could be explained by the global budget program and case payment system employed in Taiwan National Health Insurance. These payment changes may contribute to the different surgery types we observed, for example, physicians’ practices and responses to hospital payment change may vary among different diagnosis-related groups-based reimbursements (DRGs). To reduce the variation, we included provider’s attribute and clinical practice as covariates for model testing and removed those not significant covariates from the final models to improve the model. Lastly, the small effect sizes in the final model could be due to the small number of adverse outcomes, whereas a smaller effect size would require larger sample sizes, it is therefore required to determine more appropriate sample sizes for future research studies.

## Conclusions

In summary, we have demonstrated that longitudinal relationship between frailty and postoperative adverse outcomes. Frail patients experienced more short-term mortality, while pre-frailty was more strongly associated with early emergency department visits and hospital readmission. The fact that significant deterioration of adverse outcomes prediction was observed for short term prediction periods suggests that the predictive value of frailty and outcomes severity is dependent on the time frame for which the prediction is made. Frailty may have prognostic value in a shorter period of time, because frailty is a symptom that can change with aging, and short-term forecasts may be more valuable in clinical practice. Shared decision making for the planning of post-acute care decisions with caregivers and elderly frail and pre-frail surgical patients with hip fracture is necessary for enhance quality of care. In addition, a clinically usable frailty assessment instrument may have important in the prognostic counseling and care planning among older adults with hip fracture. Study highlights the importance of considering the length of the prediction period and the stability of the predictor over time when constructing a prognostic model. Lastly, the study also implies that operational criteria defining the frailty phenotype could increase its predictive validity with regard to short term adverse outcomes. Targeting the long-term relationship of frailty and adverse outcome may maximize treatment success for health outcome afterward and potentially improve older patients’ quality of life. As frailty is potentially reversible, and research in this direction might provide evidence-based guidance to prevent early emergency visit, readmission, and deaths.

## Data Availability

We were given permission to use the data from the Orthopedic study (Grant 105-CCH-HCR-115) by the CCH research committee. The datasets used for this analysis are not publicly available, as the use of data from the Orthopedic study requires the permission of the CCH research committee.
